# The American Association of Obstetricians and Gynecologists

**Published:** 1888-05

**Authors:** 


### Editorial.

### *THE AMERICAN ASSOCIATION OF OBSTETRICIANS AND GYNECOLOGISTS.*

A new national organization of specialists, who have adopted the above name for their association, was perfected in this city on the 19th of April, ultimo. It appears that a number of physicians in different portions of the country, interested in abdominal surgery, obstetrics and gynecology, have been in consultation and correspondence for a number of months past, in regard to the feasibility and propriety of organizing such a society. Sufficient encouragement was developed thereby to warrant the issuance of a circular-letter to a limited number of medical men, asking their co-operation and active support in the plan proposed. This elicited some forty favorable responses, about one-half of whom were present at the initial meeting on the day mentioned, when the organization was perfected as stated.

At this meeting, a constitution and by-laws were adopted, officers were elected, and a comprehensive working method agreed upon, looking to the future welfare, efficiency and permanency of the association. The following is the list of officers chosen:

President, William H. Taylor, Cincinnati; vice-presidents, E. E. Montgomery, Philadelphia; J. H. Carstens, Detroit; secretary, William Warren Potter, Buffalo; treasurer, X. O. Werder, Pittsburgh; executive council, Thomas Opie, Baltimore; James

H. Etheridge, Chicago; C. Cushing, San Francisco; Melancton Storrs, Hartford; Byron Stanton, Cincinnati; delegate to the Congress of American Physicians and Surgeons, James P. Boyd, Albany; alternate, Hampton E. Hill, Saco, Me.

The first annual meeting of the association was appointed to be held in Washington, September 18, 19 and 20, 1888, coincident with the meeting of the Congress of American Physicians and Surgeons, which will then and there also hold its first session.

If names speak for the success of a movement like this, or invite confidence by their weight, then we would venture the opinion that the association has been fortunate in the selection of the men who are to guide its affairs at the outset. We bespeak for this new candidate for admission to the congress a cordial welcome, and predict for it a successful career in the important fields which are its province to cultivate.

We notice, in the list of founders, the names of Drs. Thomas Lothrop, R. L. Banta and W. W. Potter, of Buffalo, all men of acknowledged excellence and professional standing, who require no encomiums from us.

Finally, we would especially congratulate the association upon its choice of Buffalo as the place of holding its inaugural meeting. It ought to be the pride of every citizen that such bodies come here for deliberation, and there should be no lack of encouragement nor faint praise to the enterprise that promotes the interests of our city, by inviting scientific or other organizations to hold their meetings within its borders.

